# The unifrac significance test is sensitive to tree topology

**DOI:** 10.1186/s12859-015-0640-y

**Published:** 2015-07-07

**Authors:** Catherine A. Lozupone, Rob Knight

**Affiliations:** Department of Medicine, University of Colorado Denver, Anschutz Medical Campus, Aurora, CO 80045 USA; Departments of Pediatrics and Computer Science & Engineering, University of California San Diego, La Jolla, CA 92093 USA

**Keywords:** UniFrac, Microbial community, Phylogenetic tree, Significance tests

## Abstract

Long et al. (*BMC Bioinformatics* 2014, 15(1):278) describe a “discrepancy” in using UniFrac to assess statistical significance of community differences. Specifically, they find that weighted UniFrac results differ between input trees where (a) replicate sequences each have their own tip, or (b) all replicates are assigned to one tip with an associated count. We argue that these are two distinct cases that differ in the probability distribution on which the statistical test is based, because of the differences in tree topology. Further study is needed to understand which randomization procedure best detects different aspects of community dissimilarities.

## Body

**Fig. 1:**
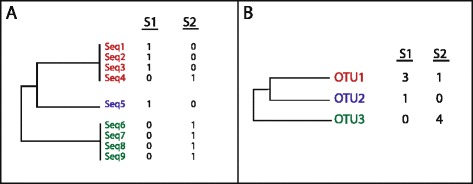
Simple representative trees representing the two different tree formats. Panel **a** shows a tree in which replicate tips, each with a count of 1, are added when the sequence is found multiple times. Panel **b** shows a tree representing the same data, but with replicate sequences represented by a single tip (e.g. as would occur if one picked OTUs and built the tree using a representative sequence for each OTU), and has a count related to each tip’s abundance in each different sample

UniFrac significance tests can be used to determine whether the types of sequences (e.g. representing bacterial 16S ribosomal RNA genes) in two different biological samples differ significantly between the samples. To do so, the sample assignments on an input phylogenetic tree are randomly re-assigned (i.e. randomizing the relationship between each tip on a tree and the sample labels), a distance between the two samples is calculated for each random dataset using either the unweighted or weighted UniFrac metric, and the fraction of the time that the true dataset has a smaller UniFrac distance between samples than the random datasets is assessed to produce a p-value [1]. In a recent paper [2], Long et al. show that the results of weighted UniFrac significance tests differ when applied to input trees in two different formats: first a tree in which replicate tips each with a count of 1 are added when the sequence is found multiple times (for example, a sequence with a count of 4 is added to the tree as 4 individual tips each with a count of 1, and a branch length of zero separating these tips from their shared parent), or second a tree in which each tip has a count related to its abundance (for example, a unique sequence that is found 4 times in a sample appears in the tree as a single tip with a count of 4) (Fig. 1). Long et al. assert that users of the UniFrac significance test should use the tool with caution, because the results can vary depending on the “arbitrary choice of input format.” They make the case that these two different tree formats are isomorphically and semantically equivalent and “merely use a different visual representation,” and that thus one should expect “any numeric calculations based on these trees to yield the same result.” We disagree strongly with these assertions

Any test based on comparing a true value to many randomizations (i.e. a Monte Carlo simulation) is performing the randomizations to empirically determine the distribution of an unknown probabilistic entity (the null distribution), so that whether the true value lies outside of this distribution can be evaluated statistically. The two different types of tree inputs described above do not change the UniFrac value of the input tree, but they do change the randomization procedure and thus the probability distribution to which the true UniFrac value is compared. The UniFrac software performs this randomization by swapping sample labels and their counts on a tip-by-tip basis using a constant tree topology, which will of course produce a different result if the tree topology is different.

An input tree in which each unique sequence is represented once with an associated count is most typically used in microbiome analysis, as this is the format that results from commonly used analysis packages such as QIIME [3] and mothur [4]. In these pipelines, sequences are first binned into Operational Taxonomic Units (OTUs) based on a percent identity threshold of their aligned 16S rRNA sequences, and a representative sequence of each OTU is used to build the tree (Fig. 1b). A 97 % identity threshold is typically used to approximate a microbial “species,” based historically on the recommendation of Stackebrandt and Goebl [5]. The case where replicate sequences are all kept in the tree (Fig. 1a) is not typically used with datasets produced with next generation sequencing, in part because they are too large to produce and manipulate computationally. It is important to note that these differences in tree topology have the potential to effect significance tests conducted with both weighted and unweighted UniFrac, as the difference in the tree topology will effect the estimate of the null distribution in both cases.

In the case where the input tree has a single representative sequence for each “species-level OTU,” the randomization procedure preserves that individual sequences from the same OTU are always assigned to a different sample together. It is thus forming the null distribution based on random assignment of microbial OTUs across samples. In contrast, using replicate tips for repeated sequences introduces the possibility that each of these tips could be randomly reassigned to a different sample and is thus forming the null distribution based on random assignment of individual sequences across samples. Further study would be needed to understand which randomization procedure, and consequently null hypothesis, may be optimal in different scenarios. However, we would recommend that in general, forming the null distribution based on a random reassignment of OTUs is more desirable than random reassignment of individual sequences that may be identical/highly related. The latter would result in 16S rRNA sequences derived from the same clonal populations of bacteria to different samples when forming the null distribution, so it is not solely testing the hypothesis that phylogenetically related but distinct bacterial taxa are in the same sample more often then chance expectation.

It is also important to note that the array of possible techniques for performing such randomizations is not limited to the methodology that we use of swapping sample labels on a constant tree topology. Another method is to instead keep the sample labels constant and to randomize the topology of the phylogenetic tree itself. This is the method used by the P test as described by Martin [6] and implemented by Schloss [7]. The P test also assesses statistical differences between the microbes in two samples using a randomization procedure, but measures distance between samples using parsimony rather than UniFrac distances [6, 7]. There are in fact many different ways to randomize a tree that could in principle be used to generate null distributions. These methods each use different ecological/evolutionary theories of how species diverge [8–11]. As is the case for weighted versus unweighted UniFrac [12], applying different randomization techniques when assessing significant differences between samples may not necessarily produce results that are “right” or “wrong”, but instead may be complementary measures that explore different aspects of how communities diverge.

Although we have considered exploring randomization methods in greater depth, in practice this has been a low priority. Such tests of significance between just two samples made sense to apply before the advent of next generation sequencing, when datasets often consisted of data from just a couple of different environmental samples. However, as the complexity of datasets has grown from just a few to thousands of samples, we have found other techniques to be more useful for statistically evaluating whether microbial composition differs across samples and whether these differences correlate with measured experimental parameters. One reason that we have found the UniFrac significance test to not be optimal for complex datasets is that pairwise tests of significance quickly loose power as the number of samples increase, because so many tests are being performed, requiring multiple comparisons corrections such as with the Bonferroni correction or False Discovery Rate (FDR) [13]. Furthermore, because significance values take into account not only the size of the biological effect but also technical parameters such as the number of sequences per sample, the practice of assessing which samples differ to the greatest degree by identifying pairs of samples that have the smallest p-value, as is done in Long et al. [2], can be misleading. The most significant p-values will not necessarily reflect the pairs with the largest effect sizes (UniFrac distances). We have thus found statistical tests that evaluate whether UniFrac distances are significantly associated with measured environmental parameters to be more powerful, for instance by applying ANOSIM [14] or Adonis [15] to UniFrac distances matrices using QIIME [3]. Another approach is to statistically compare UniFrac values to determine whether within group distances are significantly smaller than between groups distances, for instance as done to determine that gut microbiota were more similar within twins than between unrelated individuals in Turnbaugh *et al.* [16]. These types of tests are more appropriate for the larger studies that decreased sequencing cost has made increasingly common.

## References

[1] Lozupone C, Knight R (2005). UniFrac: a new phylogenetic method for comparing microbial communities. Appl Environ Microbiol.

[2] Long JR, Pittet V, Trost B, Yan Q, Vickers D, Haakensen M, Kusalik A (2014). Equivalent input produces different output in the UniFrac significance test. BMC Bioinformatics.

[3] Caporaso JG, Kuczynski J, Stombaugh J, Bittinger K, Bushman FD, Costello EK, Fierer N, Pena AG, Goodrich JK, Gordon JI (2010). QIIME allows analysis of high-throughput community sequencing data. Nat Methods.

[4] Schloss PD, Westcott SL, Ryabin T, Hall JR, Hartmann M, Hollister EB, Lesniewski RA, Oakley BB, Parks DH, Robinson CJ (2009). Introducing mothur: open-source, platform-independent, community-supported software for describing and comparing microbial communities. Appl Environ Microbiol.

[5] Stackebrandt E, Goebal BM (1994). Taxonomic note: a place for DNA-DNA reassociation and 16S rRNA sequence analysis in the present species definition in bacteriology. Int Syst Bacteriol.

[6] Martin AP (2002). Phylogenetic approaches for describing and comparing the diversity of microbial communities. Appl Environ Microbiol.

[7] Schloss PD, Handelsman J (2006). Introducing TreeClimber, a test to compare microbial community structures. Appl Environ Microbiol.

[8] Abouheif E (1998). Random trees and the comparative method: A cautionary tale. Evolution.

[9] Furnas GW (1984). The generation of random, binary, unordered trees. J Classif.

[10] Losos JB, Adler FR (1995). Stumped by trees? A generalized null model for patterns of organismal diversity. Am Nat.

[11] Maddison WP, Slatkin M (1991). Null Models for the Number of Evolutionary Steps in a Character on a Phylogenetic Tree. Evolution.

[12] Lozupone CA, Hamady M, Kelley ST, Knight R (2007). Quantitative and qualitative beta diversity measures lead to different insights into factors that structure microbial communities. Appl Environ Microbiol.

[13] Benjamini Y, Hochberg Y (1995). Controlling the False Discovery Rate - a Practical and Powerful Approach to Multiple Testing. J Roy Stat Soc B Met.

[14] Clarke KR (1993). Nonparametric multivariate analyses of changes in community structure. Aust J Ecology.

[15] Dixon P (2003). VEGAN, a package of R functions for community ecology. J Veg Sci.

[16] Turnbaugh PJ, Hamady M, Yatsunenko T, Cantarel BL, Duncan A, Ley RE, Sogin ML, Jones WJ, Roe BA, Affourtit JP (2009). A core gut microbiome in obese and lean twins. Nature.

